# Hypothesizing that, A Pro-Dopamine Regulator (KB220Z) Should Optimize, but Not Hyper-Activate the Activity of Trace Amine-Associated Receptor 1 (TAAR-1) and Induce Anti-Craving of Psychostimulants in the Long-Term

**DOI:** 10.17756/jrdsas.2016-023

**Published:** 2016-06-29

**Authors:** Kenneth Blum, Rajendra D. Badgaiyan, Eric R. Braverman, Kristina Dushaj, Mona Li, Peter K. Thanos, Zsolt Demetrovics, Marcelo Febo

**Affiliations:** 1 Department of Psychiatry & McKnight Brain Institute, University of Florida College of Medicine, PO Box 100256, 1149 Newell Dr, L4-100, Gainesville, FL 32611, USA; 2 Division of Applied Clinical Research and Education, Dominion Diagnostics, LLC., 211 Circuit Dr, North Kingstown, RI 02852, USA; 3 Division of Neuroscience-Based Therapy, Summit Estate Recovery Center, 399 Old Mill Pond Rd, Los Gatos, CA 95033, USA; 4 Division of Clinical Neurology, PATH Foundation NY, 304 Park Ave South, Floor 6, New York, NY 10010, USA; 5 Division of Nutrigenomics, LaVita RDS, 1878 W 12800S, Ste 314, Averton, UT 84085, USA; 6 Department of Psychiatry & Behavioral Sciences, Keck School of Medicine of USC, Los Angeles, CA 90033, USA; 7 Division of Neuroscience Research & Addiction Therapy, Shores Recovery & Treatment Center, Port Saint Louie, FL 34952, USA; 8 Department of Clinical Psychology and Addiction, Eötvös Loránd University, 1064 Budapest, Izabella Street 46, Hungary; 9 Department of Psychiatry and Neuroimaging, University of Minnesota, F282/2A West, 2450 Riverside Avenue South, Minneapolis, MN 55454, USA; 10 Research Institute on Addictions, University of Buffalo, 1021 Main Street, Buffalo, NY 14203, USA

**Keywords:** Trace amine-associated receptor 1 (TAAR-1), Dopamine homeostasis, Glutamatergic activity, KB220Z, Reward Deficiency Syndrome (RDS), R05166017 (TAAR1 agonist)

## Abstract

Unlike other drugs of abuse such as alcohol, nicotine, opiates/opioids, the FDA has not approved any agent to treat psychostimulant dependence. Certainly, it is widely acceptable that dopaminergic signaling is a key factor in both the initiation and continued motivation to abuse this class of stimulant substances. It is also well accepted that psychostimulants such as cocaine affect not only the release of neuronal dopamine at the nucleus accumbens (NAc), but also has powerful inhibitory actions on the dopamine transporter system. Understandably, certain individuals are at high risk and very vulnerable to abuse this class of substances. Trace-amine-associated receptor 1 (TAAR1) is a G -protein coupled receptor activated by trace amines. The encoded protein responds little or not at all to dopamine, serotonin, epinephrine, or histamine, but responds well to beta-phenylethylamine, p-tyramine, octopamine, and tryptamine. This gene is thought to be intronless. TAAR1 agonists reduce the neurochemical effects of cocaine and amphetamines as well as attenuate addiction and abuse associated with these two psychostimulants. The mechanism involves blocking the firing rate of dopamine in the limbic system thereby decreasing a hyperdopaminergic trait/state, whereby the opposite is true for TAAR1 antagonists. Based on many studies, it is accepted that in Reward Deficiency Syndrome (RDS), there is weakened tonic and improved phasic dopamine discharge leading to a hypodopaminergic/glutamatergic trait. The dopamine pro-complex mixture KB220, following many clinical trials including neuroimaging studies, has been shown to enhance resting state functional connectivity in humans (abstinent heroin addicts), naïve rodent models, and regulates extensive theta action in the cingulate gyrus of abstinent psychostimulant abusers. In this article, we are hypothesizing that KB220 may induce its action on resting state functional connectivity, for example, by actually balancing (optimizing) the effects of TAAR1 on the glutamatergic system allowing for optimization of this system. This will lead to a normalized and homeostatic release of NAc dopamine. This proposed optimization, and not enhanced activation of TAAR1, should lead to well-being of the individual. Hyper-activation instead of optimizing the TAAR1 system unfortunately will lead to a prolonged hypodopaminergic state and as such, will cause enhanced craving for not only psychoactive substances, but also other drug-related and even non-drug related RDS behaviors. This hypothesis will require extensive research, which seems warranted based on the global epidemic of drug and behavioral addictions.

## Introduction

Borowsky et al. [1] identified the trace-amine-associated receptor 1 (TAAR1). Trace-amine-associated receptor 1 (TAAR1) is a G -protein coupled receptor activated by trace amines. The encoded protein responds little or not at all to dopamine, serotonin, epinephrine, or histamine, but responds well to beta-phenylethylamine, p-tyramine, octopamine, and tryptamine. This gene is thought to be intronless. Importantly, Trace amino–acids (TAs) in the past were considered false neurotransmitters. These TAs included p-tyramine (pTyr), β-phenylethylamine (PEA), octopamine, and tryptamine are metabolites of amino acids having structural similarities of classical biogenic amines. TAs concentrations are low in the brain, but Burchett & Hicks [2] have implicated TAs in several psychiatric and neurological illnesses including schizophrenia, major depression, anxiety states, Parkinson’s disease, and even attention deficit hyperactivity disorder.

In this article, we are proposing a role for TAAR1 in “Reward Deficiency Syndrome” (RDS) involving both a hypodopaminergic and hypoglutamatergic trait/state. Certainly, the informative paper by Revel et al. [3] provided the impetus for this editorial. Along these lines, Jing & Li [4] point out that, to date, there is no FDA approved Medication Assisted Treatment for psychostimulants like cocaine abuse.

Their elegant review points out that TAAR1 knockout mice show increased sensitivity to dopaminergic activation while TAAR1 agonists reduce the neurochemical effects of cocaine and amphetamines as well as attenuating addiction and abuse associated behavioral effects of these two psychostimulants. TAAR1 agonists (R05166017) block the firing rate of dopamine in the limbic system, thereby blocking a hyperdopaminergic trait/state [5], while TAAR1 antagonists like N-(3-Ethoxy-phenyl)-4-pyrrolidin-1-yl-3-trifluromethyl-benzamide (EPPTB) [6, 7] increase agonist potency at DAD2 receptors reducing their desensitization rate, strongly suggesting a functional link between TAAR1 and D2 receptors, but in opposite directions.

It is well characterized that a hypoglutamatergic trait/state causes a reduced release of dopamine at the nucleus accumbens (NAc) due to an enhanced activation effect on GABAergic transmission in the NAc [8]. In their work, Qi et al. [8] show that electrical stimulation of the dorsal raphe (DR) and ventral tegmental area (VTA) stimulate neurons of the same reward pathway. They found that reward outcomes subsequent of DR-originating activation pathway comprised of vesicular glutamate transporter 3 (VGluT3) containing neurons that shape asymmetric synapses onto VTA dopamine neurons project onto the NAc. Moreover, they found that Optogenetic (light stimulation targeting specific neurons) VTA initiation of this projection produces AMPA-mediated synaptic excitatory currents in VTA meso-accumbens dopaminergic neurons and triggers dopamine discharge in NAc. The importance of these findings provide evidence that the DR-VGluT3 pathway to VTA employs glutamate as a neurotransmitter and is a substrate connecting the DR – an important sympathetic reward location in the brain - to VTA dopaminergic neurons. However, it is to be noted that other regions such as the PFC, amygdala, and thalamus provide a greater input to the NAc.

### Neurotransmission and the Nucleus Accumbens (NAc)

The NAc comprises medium spiny neurons (MSNs) that constitute >90% of all neurons in both the shell and core [9]. These neurons are GABAergic and contained within numerous neuropeptides limited to their projection arrangements [10]. Therefore, projections to the ventral mesencephalon are D1 encompassing and both prompt dynorphin and neurotensin; whereas, the D2 encompassing neurons projecting to the ventral pallidum both prompt enkephalin. In regards to the shell projections at the ventromedial ventral pallidum, the MSNs also comprise D1 receptors and neuropeptides involving substance P, dynorphin, and neurotensin. The lingering 5–10% of neurons in the accumbens is divided between cholinergic spiny cells and GABAergic interneurons. These two kinds of cells supply the MSNs, offering excitatory (acetylcholine) and inhibitory (GABA) tone [11]. All three cells are innervated by glutamatergic afferents from the prefrontal cortex, amygdala, thalamus, DR (most major), as well as dopamine innervation from the VTA. Interestingly, innervation from the VTA is partially GABAergic and glutamatergic; the latter is thought to be partly colocalized with dopamine [12]. This increases possible difficulties in translating the physiological and pathophysiological changes classically recognized as ‘dopaminergic’ innervation from the VTA [13].

It is now believed that dopamine alone does not produce great changes in MSN current, but potentiates or impedes the volume of glutamate to depolarize neurons. Several of these intracellular methods are well characterized and include a variation of calcium (D1) and potassium (D2) channels. Gating of glutamatergic activity can also be a lengthy process because of dopamine’s ability to control long-term excitatory synaptic plasticity. Physiologically, dopamine is at least partly colocalized with glutamate, and hence, these afferents are capable of inducing fast changes in membrane conductance [13]. Secondly, the degree to which certain glutamatergic afferents are gated by dopamine is uncertain. Glutamatergic inputs can begin from the prefrontal cortex, basolateral amygdala, hippocampus or medial thalamus [14], and as previously aforementioned, several of these afferents are at the minimum partly topographically separated between the core and shell [15]. It has been hypothesized that a balance between synaptic and non-synaptic glutamate, termed ‘glutamate homeostasis,’ plays a major role in excitatory synaptic plasticity and is a vital position of neuropathology in animal models of relapse [16]. To achieve this balance, comprehensive studies are inaccessible for the accumbens; but based on studies of the hippocampus, it is believed that the patterned manifestation of glutamate uptake transporters in the locality of excitatory synapses is liable for reducing the effect of synaptic glutamate mainly to the synaptic cleft at the position of discharge [17]. As of late, it is apparent that these transporters also guard the synaptic cleft from glutamate released from glia via calcium-dependent or cysteine–glutamate exchange mechanisms [18]. This is important, given that the glial release of glutamate maintains ~1–5 µM glutamate in the extracellular, non-synaptic space, a concentration capable of stimulating N-methyl D-aspartate (NMDA) receptors [19, 20].

While we point out the importance of other neurotransmitter (other than just dopamine) systems in addiction behavior, there is an emerging and substantial literature on the role of glutamate signaling (specifically within the NAc) in long-term addiction behavior. The NAc receives glutamate input from the VTA and PFC; both of these inputs have been well characterized in addiction behavior (specifically related to relapse). GLT-1 and xCT expression decreases following long-term drug use; GLT-1 down-regulation specifically is known to contribute to the glutamate overflow that occurs during relapse. Whether or not these changes occur as a direct result of altered dopamine signaling has not been, to our knowledge, determined. Also, it is important to note that the dopamine overflow that occurs in response to relapse behavior diminishes over time [21], whereas the glutamate response does not. Moreover, the effects of ceftriaxone on ethanol intake possible role for xCT and GLT1 isoforms seem to work by modulation of glutamine levels especially in alcohol preferring P rats [22].

## Hypothesis

This hypothesis points out that short-term hyperactivation of TAAR1 will block dopamine firing, and potentially long-term inhibition of TAAR1 will enhance dopamine-firing rate in the VTA. If we were to consider this a target for psychostimulant abuse, then it will follow the proposal of Blum et al. [23] suggesting that mild activation/optimization of TAAR1, but not hyper-activation, is proposed herein utilizing dopamine agonists. Hyper-activation of the TAAR1 will result in the blocking of mesolimbic dopaminergic reward circuitry. Although, to our knowledge, there are no long-term experiments showing that hyperactivation of the TAAR1 will increase drug seeking based on our current knowledge, we do evoke caution. We believe that our proposal is a preferred modality in the long-term treatment of all addictive behaviors or RDS.

### Amino-acid based formula for psychostimulants: rationale for KB220Z and N-Acetyl-L-Cysteine [NAC]

Blum et al. [23] proposed that D2 receptor stimulation can be accomplished with precursor amino-acids in a Prodopamine Regulator complex called KB220Z (L-tyrosine, D-Phenyalaline, L-phenylalanine, L-Tryptophane) in combination with chromium salts and *Rhodiola rosea*. They carried out a number of clinical trials using various forms of this amino-acid based formulae finding anti-craving benefit for psychostimulant abuse [24]. The Brown et al. [24] paper reported that the amino-acid based formula prevented psychostimulant relapse in out-patient driving-under-the influence (DUI) offenders. After ten-months following utilization of the amino-acid based formula, there was an overall recovery rate of 53% in cocaine abusers. In another study, Blum et al. [25] showed that the same aminoacid based formula in serious in-patient cocaine dependent subjects, significantly reduced both the withdrawal against medical advice (AMA) rate and drug hunger in a 30-day hospital treatment program. While the control group (no amino-acid based formula) had an AMA rate of 37.5% (6/16), the experimental group (amino-acid based formula) had a significantly lower (P < 0.014) AMA rate at only 4.2% (approximately a 9-fold improvement). In addition, Cold JA [26], reported on the effects of the same amino-acid based formula in the treatment of cocaine withdrawal and craving. In a double-blind placebo controlled study conducted on hospitalized patients with DSM III–R diagnosis of cocaine dependence. A significant decrease in cocaine craving occurred in the amino-acid formula compared to placebo.

This early work is supported by more recent evidence by Blum et al. [27] involving quantitative electroencephalographic (qEEG) protracted abstinence in male psychostimulant abusers. In a randomized triple–blinded placebo-controlled crossover study, the oral amino-acid based formula showed an increase of alpha and low beta wave activity in the parietal brain region. Interestingly, using t statistics, significant differences were observed in comparison to placebo, which consistently revealed a regulation of widespread theta activity in the frontal regions after week 1 and then again, after week 2 of analyses. This response was greatest in carriers of the dopamine D2 receptor A1 variant subjects (having reduced D2 receptor number by 30–40%) [28].

RDS, insensitivity, and inefficiency in the reward system has been the subject debated by many investigators and remains controversial in terms of “liking” and wanting” [29–34]. However, there may be a common neurocircuitry, neuroanatomy, and neurobiology for multiple addictions and for a number of psychiatric disorders [35]. Because of certain genetic precursors and environmental impacts (epigenetics), an insufficiency of D2 receptors may affect individuals as high-risk for several addictive, impulsive, and compulsive behaviors [36]. It is well known that alcohol and other drugs of abuse, as well as most positive reinforcers (i.e., sex, food, gambling, aggressive thrills) cause activation and neuronal release of brain dopamine and involvement of the Na^+/^K^+−^ ATPase [37]. Dopamine release can decrease negative feelings and satisfy abnormal cravings for alcohol, cocaine, heroin and nicotine, which including others, are associated with reduced dopamine activity [38]. Therefore, a formidable challenge to both scientists and clinicians in the field of substance and nonsubstance repetitive seeking behaviors is the development of compounds that can induce “dopamine homeostasis” as well as potential targets like TAAR1 [39].

### Are glutamatergic and dopaminergic pathways therapeutic targets for reward “Homeostasis”?

Glutamate and DA represent potential targets for novel treatments that modulate not only cocaine seeking behavior, but also other RDS behaviors and functional connectivity. Both substrates are affected by chronic psychostimulant administration [29, 40]. In cocaine self-administering rats, basal extracellular glutamate concentrations are reduced in the core of NAc [39], which also receives heightened PFC-evoked glutamate release [42, 43]. Evidence supports this heightened release and reduced tonic extracellular glutamate in reinstatement [43, 44]. Elevating extrasynaptic glutamate by stimulating the cysteine-glutamate exchanger using the pro-cysteine drug, N-Acetyl-L-Cysteine (NAC), has been found to reduce cue- and cocaine-primated reinstatement [43–47]. This outcome supports its development as a treatment for cocaine craving and addiction [49]. NAC restores synaptic plasticity in NAc, normalizes neuronal excitability, and glutamate transport [43, 45, 46].

**Figure 1:**
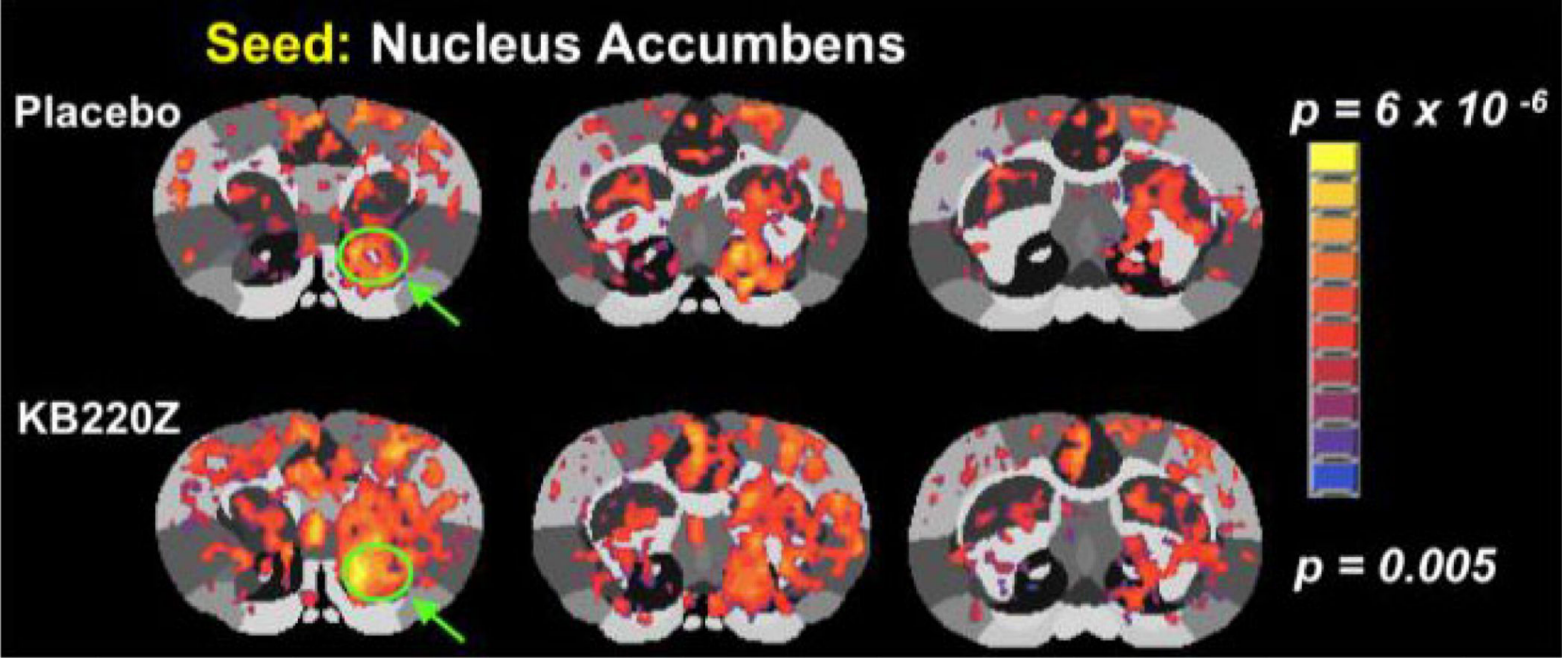
Administration of a complex (KB220Z) increases connectivity with the NAc and PFC. (unpublished data from Febo and Blum, 2015).

Additionally, it was recently shown that as cocaine intake escalates, phasic DA signaling in the ventromedial striatum is reduced [49]. The DA precursor L-DOPA was found to reduce escalated cocaine intake and restore striatal DA. This work has been furthered by the findings of Badgaiyan et al. [50] revealing that at rest, the ligand binding potential (BP) was considerably increased in the right caudate of ADHD subjects, implying lowered tonic discharge. Throughout task presentations, drastically reduced ligand BP was seen in the same region, representing improved phasic discharge. This seems reasonable and helps explain the status of RDS dopaminergic function. Consistent with this result, in human subjects, L-DOPA was observed to increase functional connectivity between midbrain and striatal regions [51]. In this regard, in unpublished work, we have examined the effects of a DA precursor complex (KB220Z a pro–dopamine complex regulator) on functional connectivity and have observed that there is a significant increase in functional connectivity strength in the PFC and NAc of rats (Figure 1). In unpublished work, we have also shown an increase in connectivity volume following seed RIOS in reward circuitry. We believe that this finding translates to increase in neuronal firing, strengthening synaptic activity. This effect would presumably benefit cocaine-addicted individuals showing reduced functional connectivity in mesocorticolimbic circuitry [52].

**Figure 2:**
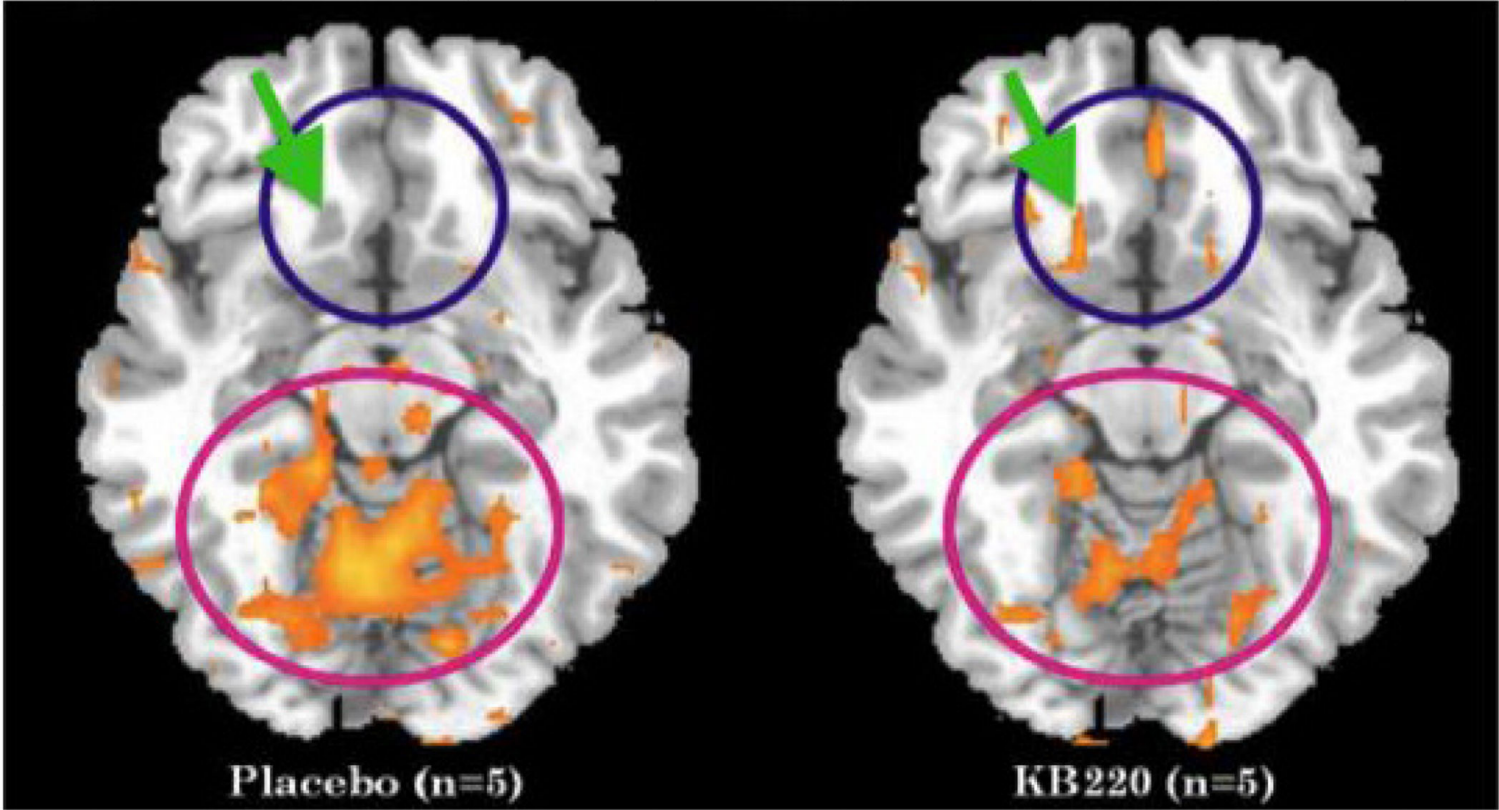
KB220Z, a DA precursor complex focused on rapid brain delivery of neurotransmitter precursors, elevates functional connectivity between regions of the accumbens and the medial orbital cortex. Arrow and blue circle are shown to emphasize increases in functional connectivity in NAc with oral KB220Z [52] (with Permission).

Key ingredients in this complex act synergistically to replenish the pool of L-DOPA and facilitate its conversion to DA. The formulation is directed at re-establishing baseline connectivity through the DA biosynthetic pathway amongst other ingredients (L-Tyrosine and pyridoxine, which provides the enzymatic co-factor pyridoxal-5’-phosphate for L-amino acid decarboxylase conversion of L-DOPA to DA) [53, 54]. Unlike just administrating L-DOPA as a precursor for dopamine, the KB220 complex provides additional ingredients that will help regulate the glutaminergic-dopaminergic systems. There is an increase, for example, of brain enkephalin due to the enkephalinase inhibition induced by D-phenylalanine; there is also glutamine as well as NAC to help balance the glutaminergic system; the formula also contains *Rhodiola rosea* that inhibits COMT activity in the synapse and MAO-A activity in the mitochondria. KB220 variant has been tested in abstinent psychostimulant abusers and found to normalize quantitative electroencephalographic (qEEG) abnormalities as well as similar effects in alcoholics and heroin addicts [27, 55]. Moreover, a preliminary double-blind cross-over study in heroin-dependent subjects shows increases in ventral striatal functional connectivity (Figure 2).

To reiterate, Willuhn et al. [49] observed increases in cocaine use and non-substance-related addictive behavior with decreased dopaminergic function. Prolonged cocaine exposure has been linked with reductions in D2/D3 receptors and was also connected to reduced cue activation in the occipital cortex and cerebellum, as reported in a new PET study by Tomasi et al. [56]. Therefore, dopamine homeostasis treatment strategies, like dopamine agonist therapy, along with glutamatergic optimization using NAC may maintain dopamine function, which seems to be a fascinating method to relapse prevention in psychoactive drug and behavioral addictions.

### Theoretical mechanisms for TAAR1 in psychostimulant abuse

It is well-known that the central nervous system (CNS) rewarding properties of ethanol, cocaine, and heroin may activate a common catechoaminegic (primary Dopamine) reward system in the mesolimbic circuitry of the brain. TAAR1, a member of the TAAR receptor family [39], is a G protein-coupled receptor (GPCR) that signals Gs to elevate intracellular cAMP levels in response to trace amino-acids. Moreover, in vitro analysis revealed a reciprocal regulation between TAAR1 and the dopamine transporter (DAT) [57]. Very importantly, in the mouse brain, it was found that TAAR1 is expressed throughout the limbic system including ventral tegmental area (VTA) and dorsal raphe nucleus (DRN).

At the moment, there remains no Food and Drug Administration (FDA) approved drug treatment for cocaine addiction. Though several neurotransmitter systems include the pharmacological effects of cocaine, dopamine plays one of the most vital roles that resolve the addiction-related behavioral effects of cocaine. Numerous pharmacological methods have been suggested to regulate the dopaminergic system to offset the abuse-related effects of cocaine. For example, antagonists that focus on diverse dopamine receptor subtypes (e.g., D1, D2, and D3) have been observed to block specific effects linked to cocaine abuse. Nonetheless, important clinical triumph has not been established, mainly due to the modest effectiveness and severe side effects, especially in the long–term treatment, including down regulation of dopaminergic receptors and potential mood changes (suicide ideation). Traditional “agonist replacement therapy” (e.g., methamphetamine or D-amphetamine for cocaine addiction; bromocyrptine) has been explored both in animals and in human subjects, and only on an acute basis, exhibits some encouraging clinical results. Conversely, anxieties about the abuse accountability of the substitute drugs per se make this method less attractive. Through this perspective, discovering substitutes that indirectly regulates the dopaminergic system could be a productive plan. Current findings strongly advise that trace amine associated receptor (TAAR) 1 could be a favorable new treatment target to fight cocaine addiction as well as other RDS behaviors.

While we have had evidence of trace amine presence in the mammalian brain for decades, their autonomous physiological responsibilities have been debatable up until the detection of TAARs. Specifically, TAAR 1 has been duplicated from both rodent and primate brains and symbolizes the most extensively studied TAAR as of yet. Along with the help of genetically modified mice, we now understand that TAAR 1 contributes to the regulation of dopaminergic function. TAAR 1 knockout mice express a behavioral phenotype of supersensitivity to dopaminergic stimulation, with augmented behavioral reaction to amphetamines. In contrast, certain brain overexpression of TAAR 1 produces a behavioral phenotype that is hyposensitive to amphetamines. These outcomes indicate a functional variation of dopaminergic system by TAAR 1 [7], which increases the likelihood of pharmacologically directing TAAR 1 for the management of psychiatric disorders whose pathophysiology includes dysregulation of the dopaminergic system, such as schizophrenia, depression and drug addiction [3, 5]. However, in this hypothesis we are suggesting the complete opposite to what is currently believed about the role of TAAR1 in all addictive behaviors.

Importantly, it was discovered that TAAR 1 agonists exhibit extremely encouraging antipsychotic-like outcomes in preclinical studies and show excellent therapeutic profiles than current antipsychotics because they subdue feeding and reduce body weight in animals; hence, lacking the main unfavorable effect of weight gain as seen when taking some antipsychotics. These data align with the view that TAAR 1 agonists could functionally regulate the dopaminergic system and therefore, it is an expected hypothesis that these compounds may also modify stimulant abuse. However, we do not agree that this approach over the long-term would be beneficial and in fact, it may be harmful [23].

TAAR 1 agonists can stop addiction-linked outcomes of cocaine use in rats [58, 59]. In the Pei et al. [58] study, a TAAR 1 partial agonist RO5203648 and a TAAR 1 full agonist RO5256390 [3, 5], considerably lowered context cue- and cocaine-stimulated reinstatement to cocaine seeking behavior, a broadly used animal model of cocaine relapse. Neurochemical studies discovered that RO5203648 prevented cocaine-induced dopamine overflow in the nucleus accumbens, a key brain region in drug addiction.

The Thorn et al. [59] study employed more extensive behavioral analyses and examined the impact of a TAAR 1 partial agonist RO5263397 [5] on several abuse-related effects of cocaine. It was discovered that RO5263397 appreciably decreased both context cue- and a priming dose of cocaine-associated reinstatement of cocaine seeking behavior, which is constant with the effects of using other TAAR 1 agonists (RO5203648 and RO5256390) [58]. Additionally, RO5263397 was seen to weaken notably the expression of cocaine-stimulated behavioral sensitization and habituated place partiality, two frequently used models for the study of drug-linked behavioral neuroplasticity. With RO5263397, rats terminated cocaine use earlier when the capacity for attaining cocaine was increasingly amplified, signifying that RO5263397 reduced the incentive of cocaine consumption in animals.

Finally, we are cognizant of other important systems now being recognized such as the Neurokinin-1 (NK1) receptor system. While additional work is required in this fruitful area of research and understanding that that SP/NK1 receptor system is involved in processing of positive incentive anticipation and plays a role in accentuating positive valence in association with the primary dopaminergic pathways in the reward circuit, any definitive conclusions of our hypothesis and even other endogenous systems linked to appropriate psychostimulant therapy must await additional scientific evidence [60].

### Our hypothesis and conclusion

While these reviewed data are noteworthy because they examined the concept that pharmacologically regulating TAAR 1 can decrease cocaine addiction utilizing animal representations with suitable translational and predictive scores, we are proposing that TAAR1 agonists may be beneficial only in the short–term, but should not be utilized in long-term treatment in humans due to interference with natural dopaminergic regulation.

Moreover, KB200z, is a formulation containing both a D1 agonist and NAC. We hypothesize that this compound should lead to a normalized and homeostatic release of NAc dopamine. NAC has been well studied in the treatment of cocaine relapse and has shown promising therapeutic results. However, others have demonstrated its effects to be due to normalization of xCT and basal glutamate levels. Thus, KB200z may be effective in the treatment of addiction not through regulation of the dopamine but rather the glutamate system. We must await these anticipated studies to help clarify our hypothesis.

Along these lines, we are further hypothesizing that the dopamine pro-complex mixture known as KB220z (a putative indirect dopamine agonist containing NAC) may induce its action on resting state functional connectivity. For example [54], by actually balancing (optimizing) the effects of TAAR1 on the glutamatergic system allowing for optimization of this system, should lead to a normalized and homeostatic release of NAc dopamine causing well-being of the individual. Hyper-activation instead of optimizing the TAAR1 system unfortunately will lead to a prolonged hypodopaminergic state and as such, will cause enhanced craving for not only psychoactive substances, but also other drug-related and even non-drug related RDS behaviors.

While others have suggested that it is important to balance both glutaminergic and dopaminergic systems to treat addiction [61] unlike us in this hypothesis, they have not provided any clear pathway to achieve this laudable goal. Thus, we believe that this hypothesis contributes to the literature.
